# Effectiveness of Virtual Reality in Nursing Education: Meta-Analysis

**DOI:** 10.2196/18290

**Published:** 2020-09-15

**Authors:** Feng-Qin Chen, Yu-Fei Leng, Jian-Feng Ge, Dan-Wen Wang, Cheng Li, Bin Chen, Zhi-Ling Sun

**Affiliations:** 1 School of Nursing Nanjing University of Chinese Medicine Nanjing China

**Keywords:** virtual reality, nursing education, meta-analysis

## Abstract

**Background:**

Virtual reality (VR) is the use of computer technology to create an interactive three-dimensional (3D) world, which gives users a sense of spatial presence. In nursing education, VR has been used to help optimize teaching and learning processes.

**Objective:**

The purpose of this study was to evaluate the effectiveness of VR in nursing education in the areas of knowledge, skills, satisfaction, confidence, and performance time.

**Methods:**

We conducted a meta-analysis of the effectiveness of VR in nursing education based on the Cochrane methodology. An electronic literature search using the Cochrane Library, Web of Science, PubMed, Embase, and CINAHL (Cumulative Index to Nursing and Allied Health Literature), up to December 2019 was conducted to identify studies that reported the effectiveness of VR on knowledge, skills, satisfaction, confidence, and performance time. The study selection and data extraction were carried out by two independent reviewers. The methodological quality of the selected studies was determined using the Cochrane criteria for risk-of-bias assessment.

**Results:**

A total of 12 studies, including 821 participants, were selected for the final analysis. We found that VR was more effective than the control conditions in improving knowledge (standard mean difference [SMD]=0.58, 95% CI 0.41-0.75, *P*<.001, *I^2^*=47%). However, there was no difference between VR and the control conditions in skills (SMD=0.01, 95% CI –0.24 to 0.26, *P*=.93, *I^2^*=37%), satisfaction (SMD=0.01, 95% CI –0.79 to 0.80, *P*=.99, *I^2^*=86%), confidence (SMD=0.00, 95% CI –0.28 to 0.27, *P*=.99, *I^2^*=0%), and performance time (SMD=–0.55, 95% CI –2.04 to 0.94, *P*=.47, *I^2^*=97%).

**Conclusions:**

The results of this study suggest that VR can effectively improve knowledge in nursing education, but it was not more effective than other education methods in areas of skills, satisfaction, confidence, and performance time. Further rigorous studies with a larger sample size are warranted to confirm these results.

## Introduction

With the rapid development of information technology and shortages of nurse workforce, a transformation of nursing education is needed to prepare nursing students for evolving and complex health care environments [1-3]. In US nursing schools, 75,029 qualified applicants for bachelor’s degrees and nursing postgraduate courses were rejected in 2018 due to an insufficient number of faculty, clinical sites, classroom space, clinical preceptors, and budget constraints [4].

The ultimate goal of nursing education is to promote the application of theoretical knowledge in clinical practice [5]. However, limited clinical practice time affects the opportunity for students of having clinical experience with real patients [6]. This lack of clinical practice, which prepares students for the real clinical environment, can contribute to nursing procedure errors that compromise the safety of patients [7]. Narrowing the gap between theory and practice during the educational process is necessary, but poses several challenges to nursing educators [8]. In this scenario, to guarantee the quality and safety of nursing education, educators have adopted various teaching strategies including simulation experience for students [9].

Simulation has been shown to be a valuable teaching-learning strategy to support the changing world of nursing education and to help optimize the teaching process [10-12]. As the National Council of State Boards of Nursing stated, simulation is a key component of nursing education [13]. The use of simulation as a nursing education tool is becoming increasingly common, providing students with realistic opportunities to practice skills learned in theory [14]. Through simulation, students have a variety of practical opportunities to repeat clinical scenarios and make immediate decisions and reflections [15].

With the development of simulation technology, the virtual world was discovered—initially used in military and medical science and later, in medical education [16,17]. Virtual reality (VR) is the use of computer technology to create an interactive three-dimensional (3D) world in which users have a sense of spatial presence [18]. It provides a first-person active learning experience through different degrees of immersion, or, in other words, the real perception of the digital world and the ability to interact with objects and/or perform a series of actions in this digital world [19,20]. VR is highly conducive to clinical and procedure-focused training by enabling simulation [21]. VR simulation refers to the use of a variety of immersive, highly visual, 3D characteristics to replicate real-life situations and health care procedures, incorporating physical or other interfaces such as a computer keyboard, a mouse, speech/voice recognition, motion sensors, or haptic devices [22]. Virtual simulation refers to the involvement of real people operating simulated systems via a computer screen (virtual, that is, as the situation is not physical or in real time), and may include surgical simulators used for on-screen procedural training, usually integrated with haptic devices to interact with the system [18]. In general, VR can make simulation become an effective supplemental tool for teaching [22,23].

As VR technology advances and becomes increasingly affordable, nursing education is being transformed [24]. VR has gained increasing attention in the field of nursing education and been used to teach many nursing concepts including leadership, communication, decision-making, critical thinking, inclusivity, health appraisal, and disaster triage [25,26]. The use of VR in simulations allows repetitive, hands-on training to develop cognitive and skill mastery among nursing students, which are usually defined as the measure of participants’ understanding of concepts and the ability of a participant to demonstrate a procedure or technique, respectively [8,27]. Additionally, VR simulations can give nursing students the opportunity to practice skills in a safe environment without risk to patients [28]. In a study, 98% of the participating students recommended virtual simulation for future use in nursing education [29].

Although the use of VR has many advantages, some researchers have reported that VR is not more effective than other traditional methods on some outcomes such as knowledge and performance scores [30,31]. There are still some inconsistencies on the effectiveness of VR among studies. Up to date, meta-analyses on the effectiveness of VR have been conducted in some areas of medicine and education [32,33]. However, to the best of our knowledge, there is no meta-analysis evaluating the effectiveness of VR in nursing education. Only one article systematically reviewed and evaluated the effectiveness of VR without meta-analysis, focusing on the effectiveness of VR simulation compared to other simulated methods on clinical psychomotor skills for pre-registration nursing students [34]. Therefore, there is a need to determine the effectiveness of VR in nursing education. The aim of this study was to perform a meta-analysis of the effectiveness of VR use on knowledge (participants’ understanding of concepts), skills (ability of participants to demonstrate a procedure or technique), satisfaction (participants’ perception with VR learning intervention), confidence (self-confidence in learning content and process), and performance time (time taken on the test task) in nursing education.

## Methods

This meta-analysis was conducted according to the PRISMA (Preferred Reporting Items for Systematic Review and Meta-Analyses) guidelines [35].

### Search Strategy

An electronic literature search was carried out in the Cochrane Library, Web of Science, PubMed, Embase, and CINAHL (Cumulative Index to Nursing and Allied Health Literature) from their inception to December 2019. The search strategies used in PubMed, Embase, and the Cochrane Library are listed in the Multimedia Appendix 1. Slightly modified search strategies were used in the other databases. Additionally, we manually examined reference lists of the selected articles to retrieve other relevant publications. Two investigators conducted searches independently, and EndNote software was used to import and manage selected documents.

### Inclusion Criteria

This study included randomized controlled trials (RCTs) or trials employing quasi-experimental randomized design, including those in the form of dissertations and conference papers, based on the PICO (Population–Intervention –Comparison–Outcome) method. In this study, the PICO elements were as follows:

- Population: pre-/post-registration nursing students or nursing staff
- Intervention: all kinds of VR education methods
- Comparison: traditional education methods (including presentations, classes, written instructions, etc) and non-VR simulation methods (including high/low fidelity simulation, mannequin-based simulation, etc)
- Outcomes: knowledge, skills, satisfaction, confidence, and performance time

### Data Extraction

Two reviewers (FQC and YFL) independently extracted information based on preset standards, including authors, publication date, nation, sample size, participants type, research project, intervention regimens, and outcomes.

### Risk-of-Bias Assessment

Two reviewers (FQC and YFL) assessed the studies’ quality independently by referring to the Cochrane Handbook for Systematic Reviews of Interventions [36], which includes 7 domains corresponding to a specific type of bias: (1) random sequence generation (selection bias); (2) allocation concealment (selection bias); (3) blinding of participants and personnel (performance bias); (4) blinding of outcome assessment (detection bias); (5) incomplete outcome data (attrition bias); (6) selective reporting (reporting bias); and (7) other biases. A judgement of “low risk,” “high risk,” or “unclear risk” of bias was assigned to each domain. When disagreements between reviewers could not be resolved through discussion, two additional reviewers (ZLS and JFG) made the final decision.

### Data Synthesis and Analysis

The meta-analysis was conducted using RevMan 5.3 [37], a desktop version of Review Manager software used for Cochrane intervention and flexible reviews. For continuous data, we reported standard mean difference (SMD) with 95% confidence intervals. In each analysis, *I*^2^ was used to measure the statistical heterogeneity among studies. According to the values of *P* and *I*^2^, the fixed-effect model (*P*>.1, *I*^2^<50%) or random-effects model (0<*P*<.1, *I*^2^≥50%) were selected [38].

## Results

### Results of the Literature Search

**Figure 1:**
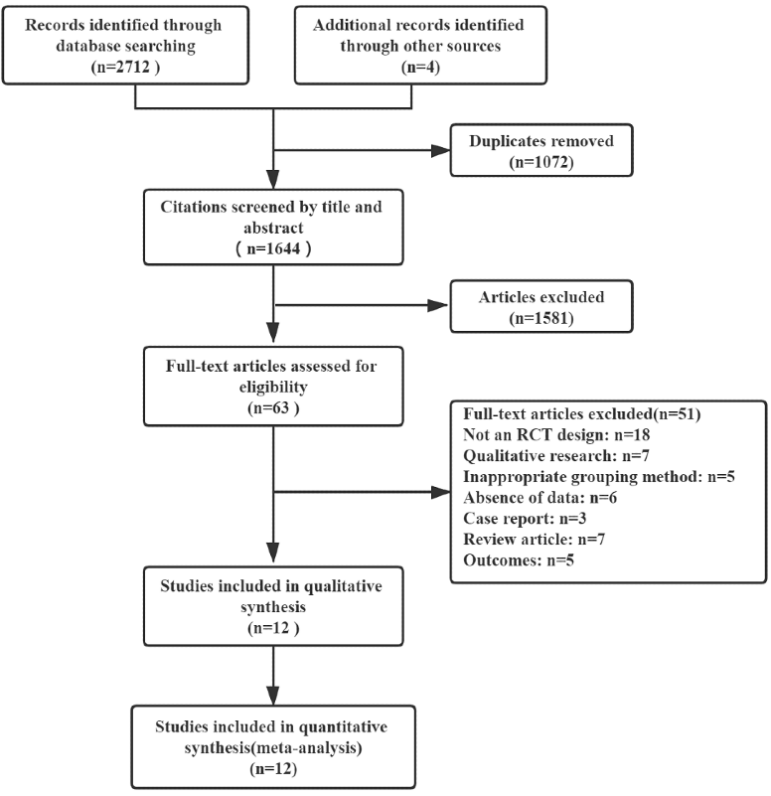
Flowchart of the study selection process. RCT: randomized controlled trial.

A total of 2716 potential studies were identified from 5 databases (n=2712) and relevant references (n=4). After removing 1072 duplicates, the remaining articles were reviewed and those that did not meet the inclusion criteria were excluded. A total of 1644 articles were screened by title and abstract, of which 1581 articles were excluded. A total of 63 full-text articles were downloaded and assessed, from which 51 were excluded. Finally, 12 studies, including 821 participants, were selected for this study. A flow chart of the study selection process is presented in Figure 1.

### Study Characteristics

Studies included trials conducted in 7 countries: United States [31,39-42], Turkey [43], Canada [44], Korea [45], Singapore [46], Portugal [47], and China [48,49]. Two trials adopted a 3-arm group design [42,45], while 10 trials used a 2-arm group design. Sample sizes ranged from 20 to 172 participants. In all trials, participants were nursing students, except for one study in which participants were nursing staff [49]. Six of 12 trials compared VR education with traditional education [31,39,41,42,48,49], while the remaining trials compared VR education with other simulation types including fidelity manikin [44,47], mannequin-based simulation [40,45,46], and plastic model [43]. The characteristics of the participants, intervention details, and outcome measures are presented in Table 1. Supplementary information of intervention in experimental and control conditions is shown in Multimedia Appendix 2.

**Table 1:** Characteristics of the 12 included studies.

Author (year), country	Type of participant	Research project	Number of participants			Outcomes
			Total (number of groups)	Experimental group (VR^a^)	Control group (condition)	
Bryant et al (2015) [31], USA	Nurse practitioner students	Advanced health assessment	60 (2)	22	38 (traditional education)	Satisfaction, confidence
Butt et al (2018) [39], USA	Junior level nursing students	Urinary catheterization	20 (2)	10	10 (traditional education)	Performance time
Cobbett and Snelgrove-Clarke (2016) [44], Canada	Third-year nursing students	Maternal -newborn nursing	56 (2)	27	28 (non-VR simulation)	Self-confidence
Haerling (2018) [40], USA	Fifth- and sixth-quarter associate degree in nursing students	Nursing care of patients with chronic obstructive pulmonary disease	28 (2)	13	15 (non-VR simulation)	Knowledge assessment, performance scores, satisfaction, and self-confidence
Ismailoglu and Zaybak (2018) [43], Turkey	Second-year nursing students	Intravenous catheter insertion	65 (2)	33	32 (non-VR simulation)	Knowledge assessment, skill scores, self-confidence scores
Jung et al (2012) [45], Korea	First-year nursing students	Intravenous injection	114 (3)	38	38 (non-VR simulation) and 38 (VR plus non-VR simulation)	Procedure score, satisfaction, performance time
Leflore et al (2012) [41], USA	Senior nursing students	Care of pneumonia and cystic fibrosis exacerbation	93 (2)	46	47 (traditional education)	Knowledge assessment
Liaw et al (2014) [46], Singapore	Senior nursing students	Assessing and managing deterioration	61 (2)	31	30 (non-VR simulation)	Performance scores
Padilha et al (2019) [47], Portugal	Second-year nursing students	Respiratory process in relation to ineffective airway clearance and hypoxia	42 (2)	21	21 (non-VR simulation)	Knowledge assessment, satisfaction
Smith et al (2018) [42], USA	Senior nursing students	Decontamination training	172 (3)	59 (immersive VR) 58 (desktop VR)	55 (traditional education)	Knowledge assessment
Tsai et al (2008) [49], China	Novice nurses	Port-A cath injection	82 (2)	42	40 (traditional education)	Knowledge assessment
Gu et al (2017) [48], China	Second-year students	Course of fundamental of nursing	28 (2)	14	14 (traditional education)	Knowledge assessment

^a^VR: virtual reality.

### Risk of Bias

**Figure 2:**
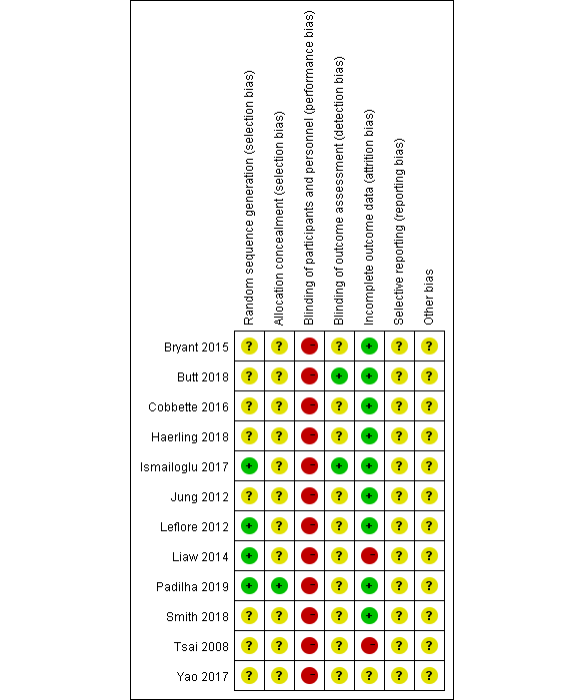
Risk of bias analysis of each included study.

**Figure 3:**
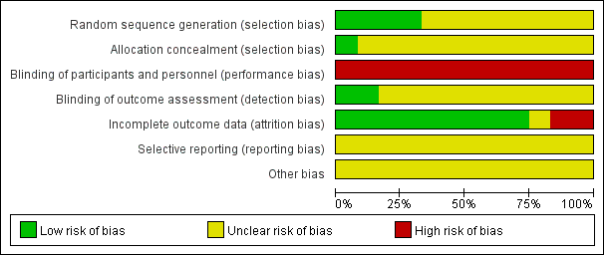
Overall risk of bias analysis of included studies.

Based on the Cochrane criteria, a risk-of-bias assessment is presented in Figures 2 and 3. Four of 12 studies reported randomized methods in detail [41,43,46,47], while the remaining 8 trials did not provide the methods of sequence generation. None of the trials provided concealment methods, except for one that reported the use of anonymization [47]. In all trials, no blind method was used on participants due to the particularity of the intervention methods. Two trials reported employing blinding of assessors [39,43]. Additionally, 2 studies mentioned dropouts without detail on handling information [46,49].

### Results of the Meta-analysis

#### Knowledge

**Figure 4:**
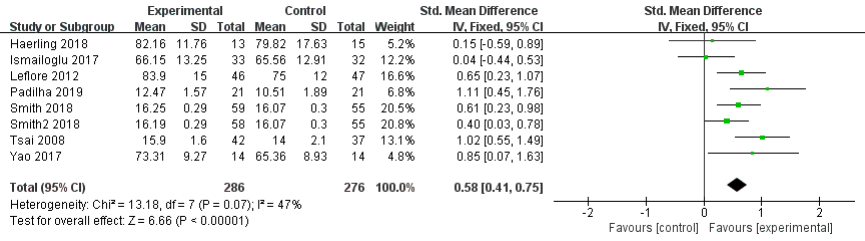
Forest plot of virtual reality on knowledge.

A total of 7 studies reported knowledge scores as the outcome [40-43,47-49]. The results indicated that VR education can improve knowledge of participants more effectively than the control conditions (SMD=0.58, 95% CI 0.41-0.75, *P<*.001, *I*^2^=47%, Figure 4).

#### Skills

**Figure 5:**
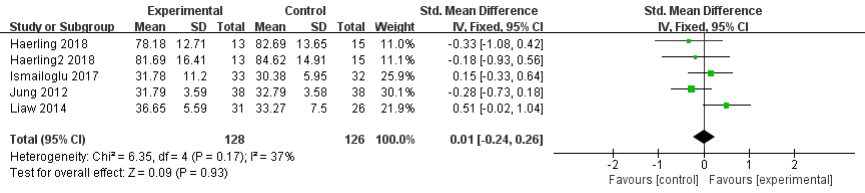
Forest plot of virtual reality on skills.

A total of 5 trials used skills as the outcome measure [40,42,43,45,46]. The results indicated that there was no significant difference between VR education and other education methods on skills enhancement (SMD=0.01, 95% CI –0.24 to 0.26), *P=*.93, *I*^2^=37%; Figure 5).

#### Satisfaction

**Figure 6:**
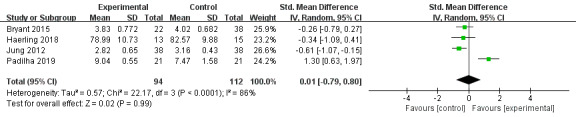
Forest plot of virtual reality on satisfaction.

A total of 4 articles reported participants’ satisfaction scores [31,40,45,47]. Participants in VR groups showed no difference when compared to control groups (SMD=0.01, 95% CI –0.79 to 0.80, *P=*.99, *I*^2^=86%). High heterogeneity was found. The leave-one-out method was used to carry out sensitivity analysis, and the random-effects model was adopted. One trial [47] caused significant heterogeneity, showing VR education is more satisfactory to participants than the control conditions (SMD=1.30, 95% CI 0.63-1.97, *P=*.001; Figure 6).

#### Confidence

**Figure 7:**
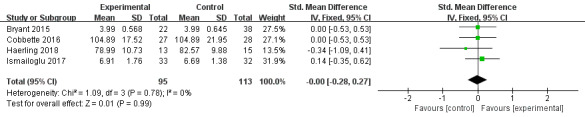
Forest plot of virtual reality on confidence.

A total of 4 studies reported confidence results [31,40,43,44] and showed no statistical difference between VR education and other education methods (SMD=0.00, 95% CI –0.28 to 0.27, *P=*.99, *I*^2^=0%; Figure 7).

#### Performance time

**Figure 8:**
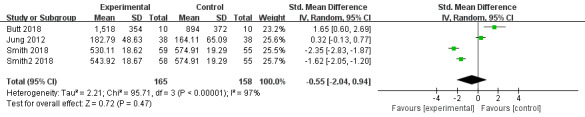
Forest plot of virtual reality on performance time.

Performance time was employed as an outcome measure in 3 trials [39,42,45]. There was no significant difference between the experimental and control groups (SMD=–0.55, 95% CI –2.04 to 0.94, *P=*.47, *I*^2^=97%]. Heterogeneity in this outcome was high. Therefore, the random-effects model was used, and the sensitivity analysis was carried out by using the leave-one-out method. Nevertheless, heterogeneity remained significant even when removing one study at a time (Figure 8).

## Discussion

This meta-analysis assessed the effectiveness of VR simulation methods in nursing education. We found that VR education methods can improve the knowledge of nursing students. However, there was no difference between VR and other education methods on the outcomes of skills, satisfaction, confidence, and performance time.

A total of 12 trials with 821 participants were included in the meta-analysis. All studies used VR education as the interventions in experimental groups, and education methods in control groups including traditional education, high/low fidelity manikin, mannequin-based simulation, and plastic model. Among the 12 studies, 4 trials reported random sequence generation. Only 1 study described the allocation concealment; 2 studies reported the blindness of outcome assessment. In addition, blinded interventions of students and educators were not possible because of the particularity of the VR education method. In general, the overall risk of bias of the included studies was judged to be unclear due to lack of information.

### Knowledge

For the outcome of knowledge, VR education showed more effectiveness on nursing education than traditional education or other simulation education methods. A qualitative study on VR use in nursing education also concluded that, through the concrete experience of the virtual patient simulation and the reflection tool, students could understand what they were taught and how to utilize the new knowledge [50]. Additionally, a previous study, which focused on virtual reality for health professions education, indicated that VR with higher interactivity showed more effectiveness for knowledge [21]. These studies support the fact that an interactive learning environment encourages students to establish connections between concepts [51]. Most of the studies included in our meta-analysis used interactive VR education methods, which could explain the results.

### Skills

Our results found no significant difference between VR education and other education methods for the outcome of skills, which seems to be in line with a previous systematic review [34]. The review concluded that virtual reality groups performed comparably to simulation groups in skill performance scores and skill success rate [34]. In our study, all the included trials that reported skills employed other simulation education methods in control groups. Similarly, we concluded that VR was not more effective in improving skills than other simulation methods in nursing education. A possible reason for these results is that there is a gap between completing virtual cases and real practice. Nursing skills learned on a virtual platform may not be transferable to real situations effectively because of the immaturity of VR technology [48].

### Satisfaction

There was no significant difference on participants’ satisfaction between VR education and education methods in control groups. High heterogeneity was found. Through sensitive analysis, we found that 1 of the 4 included studies showed that VR was more satisfactory [47]. In one trial conducted in 2012, some participants pointed out the immaturity of VR technology affecting users’ satisfaction [45]. In contrast, 2 studies in recent years showed no difference between the 2 groups [31,40]. Thus, we consider that participants’ satisfaction with VR education may vary according to technical conditions. Although in the 21st century nursing students had already shown high levels of usefulness, ease, and intention to use clinical VR simulation, VR is not widely used in nursing education [52]. With the progress of technology, VR can better satisfy the users. However, further research is needed to confirm our results.

### Confidence

The results in confidence indicated no difference between experimental and control conditions. VR could not enhance the confidence of participants more effectively than control conditions, which was consistent with a previous study from Korea [53]. When VR was used for operation exercises, it was often necessary to use a mouse at the same time [53]. Thus, the operation method is more difficult in VR when compared with other simulations such as the manikin.

### Performance Time

We also conducted a meta-analysis of performance time. The results suggested that VR was not more effective on reducing performance time than other educational methods. We found large heterogeneity among studies, even when a sensitivity analysis was conducted by using the leave-one-out method. The observed heterogeneity may be due to the different research designs of the selected studies, such as operation projects, VR devices, and education methods in control groups. One study on the effectiveness of VR endoscopy simulation training analyzed performance time with sufficient data and found no difference between VR and control groups; however, the quality of the evidence was very low [54]. In contrast, a study conducted in clinical medicine found that VR can help operators shorten performance time [55]. Therefore, more experiments are needed in the future to study the effectiveness of VR on performance time in nursing education.

### Strengths and Limitations

Our study has the following strengths. First, our study is the first meta-analysis assessing the impact of VR on nursing education. Second, to assess the effectiveness of VR education, we evaluated 5 outcome measurements—knowledge, skills, satisfaction, confidence, and performance time—which can probably provide reference for nursing education.

There are also some limitations in our study. First, we only included articles published in English, which may affect the results of meta-analysis. Second, some of the included studies failed to provide the details of sequence generation, allocation concealment, and blinding methods. Third, we included 12 studies that have different interventions in control groups, which may cause significant heterogeneity among the studies.

### Conclusions

This meta-analysis provides a comprehensive evaluation of the use of VR on nursing education. We found that VR education methods can improve nursing students’ knowledge. However, for the outcomes of skills, satisfaction, confidence, and performance time, there seems to be no difference between VR and other education methods. In general, the use of VR should be considered to enhance knowledge and as a complement of other simulation strategies to improve the quality and safety of clinical practice. However, the heterogeneity and risk of bias among the included studies should be taken into consideration. Rigorously designed large-scale studies are required to further confirm the results in this review.

## Appendix

Multimedia Appendix 1 Search strategies of PubMed, Embase and the Cochrane Library.

Multimedia Appendix 2 Supplementary information of intervention in experimental and control groups.

## Abbreviations

CINAHL: cumulative index to nursing and allied health literature
PICO: Population–Intervention–Comparison–Outcome
PRISMA: Preferred Reporting Items for Systematic Review and Meta-Analyses
RCT: randomized controlled trials
VR: virtual reality
