# Supplementary material for: Effectiveness of Virtual Reality in Nursing Education: Meta-Analysis
Source: J Med Internet Res. 2020 Sep 15;22(9):e18290. doi: 10.2196/18290 (PMC7525398; doi:10.2196/18290)
Supplement: Multimedia Appendix 1 [file jmir_v22i9e18290_app1.docx]

## Multimedia Appendix 1:

## Search strategies

PubMed

#1. Virtual Reality[Mesh]

#2. Patient Simulation[Majr]

#3. ((("virtual reality"[Title/Abstract]) OR (''patient simulat*"[Title/Abstract])) OR ("virtual patient*"[Title/Abstract])) OR ("virtual simulation"[Title/Abstract])

#4. #1 OR #2 OR #3

#5. Education, Nursing[Mesh]

#6. (("nurs* education"[Title/Abstract]) OR ("education of nursing"[Title/Abstract])) OR ("nursing, education"[Title/Abstract])

#7. #5 OR #6

#8. #4 AND #7

Embase

#1. 'virtual reality'/exp

#2. 'virtual reality':ab,ti OR 'patient simulat*':ab,ti OR 'virtual patient*':ab,ti OR 'virtual simulation':ab,ti

#3. #1 OR #2

#4. 'nursing education'/exp

#5. 'nurs* education':ab,ti OR 'education of nursing':ab,ti OR 'nursing, education':ab,ti

#6. #4 OR #5

#7. #3 AND #6

The Cochrane Library

#1. MeSH descriptor: [virtual reality] this term only

#2. ("virtual reality"):ti,ab,kw OR (''patient simulat*"):ti,ab,kw OR ("virtual patient*"):ti,ab,kw OR ("virtual simulation"):ti,ab,kw

#3. #1 OR #2

#4. MeSH descriptor: [Education, Nursing] this term only

#5. ("nurs* education"):ti,ab,kw OR ("education of nursing"):ti,ab,kw OR ("nursing, education"):ti,ab,kw

#6. #4 OR #5

#7. #3 AND #6
