# Supplementary material for: Effectiveness of Virtual Reality in Nursing Education: Meta-Analysis
Source: J Med Internet Res. 2020 Sep 15;22(9):e18290. doi: 10.2196/18290 (PMC7525398; doi:10.2196/18290)
Supplement: Multimedia Appendix 2 [file jmir_v22i9e18290_app2.doc]

**Multimedia Appendix 2**

Table 2 Supplementary information of intervention in experimental and control groups.

| Study(author/year) | Experimental group | Control group |
| --- | --- | --- |
| Bryant 2015 | (A)VR: virtual clinical simulation product (Digital Clinical Experience ^TM^) involved the assessment of a single virtual patient across multiple systems-based modules by students in the intervention group. Participants pose questions via typed text, direct the digital Standardized Patient’s actions, engage in empathetic or educational interactions with the DSP through typed speech, and use virtual equipment and instruments to perform a full physical assessment. | (B)Traditional education: Standard course activities, including assigned readings, faculty lectures, videos, unfolding case studies, quizzes, and examinations. |
| Butt 2018 | (A)VR: a game-based virtual reality system, in which participants were assisted into the Oculus Rift headgear and wearable haptics including sensory gloves, and synchronize and calibrate the equipment to each subjects’ movement. | (B)Traditional education: One-hour practice session, supervised by an experienced faculty member and supplies were provided to practice urinary catheterization on a partial task trainer with immediate expert feedback on performance. |
| Cobbette 2016 | (A)VR: a virtual clinical simulation (vSim®) for Nursing co-developed by Laedral and Wolters Kluwer Health from Lippincott, which uses a combination of computer multimedia with a central video or virtual world to produce interactive learning activities mediated by the learner. | (B)Non-VR simulation: F2F high-fidelity manikin clinical simulation. |
| Haerling 2018 | (A)VR: a web-based, commercially available virtual simulation (vSim) that can allow participants in the virtual simulation groups interacted independently with the computer-based simulated patient. | (B)Non-VR simulation: Mannequin-based simulation, using live, professional facilitated, mannequin-based simulation. |
| Ismailoglu 2017 | (A)VR: a virtual intravenous simulator system that uses a haptic device, which requires physical contact between computer and user, a software program, a desktop or laptop computer, and a virtual IV anatomical viewer. | (B)Non-VR simulation: plastic intravenous injection arm model, using an adult-sized plastic arm with multivascular system designed for IV injection training, containing visible, palpable venous vessels. |
| Jung 2012 | (A)VR: a virtual reality techniques (IV sims) which use haptic devices that provide a sense of resistance during venipuncture and realistic three-dimensional photographic images observed using polarized glasses in simulated environments. | (B)Non-VR simulation: Mannequin human arm (IV arms), using a model for practical exercises focusing on securing veins, consisting of skin and veins that are nearly identical to those of humans |
| Leflore 2012 | (A)VR: a virtual patient trainer using Virtual Pediatric Patients (VPPs) and a Virtual Pediatric Unit (VPU) by using Unreal Engine 3 which included orientation, mini-games, virtual patient experience, and debrieﬁng/feedback. | (B)Traditional education: 3-hour pediatric respiratory lecture by faculty. |
| Liaw 2014 | (A)VR: a virtual patient simulation developed at National University of Singapore. This single user interactive multimedia simulation was created using Flash software and run on a secure server. Learners choose to participate in any scenarios by clicking on the patient’s day of admission to interact with virtual patient. | (B)Non-VR simulation: Mannequin-based simulation led by a trained simulation facilitator. |
| Padilha 2019 | (A)VR: a clinical virtual simulation that use digital and virtual technology to recreate reality using virtual patients depicted on a computer touchscreen. Clinical virtual simulation uses virtual patients in dynamic and immersive clinical environments ranging from prehospital environments to environments in the community. | (B)Non-VR simulation: Low-fidelity simulator and realistic environment (pedagogical strategies that were already used in the nursing school), guided throughout by the regular subject teacher. |
| Smith 2018 | (A)VR1: immersive VR that is based on motiontracked, stereoscopic 3D head-mounted display (HMD) and motion-tracked hand controllers.  (B)VR2: desktop VR and presented the same 3D environment monoscopically on a computer screen with mouse-and-keyboard controls. | (C)Traditional education: written instructions. |
| Tsai 2008 | (A)VR: a virtual reality port-A cath simulation by using 3D computer graphics, interactive circumscribed and external hardware controls linking to the desk top computer, provide a humanized computer interface to immerse a user in an artiﬁcial environment and allow real-time sensory interaction. | (B)Traditional education: nursing class. |
| Yao 2017 | (A)VR: virtual simulation (vSIM) for Nursing platform developed through a collaboration among Wolters Kluwer Health, Laerdal Medical, and the National League for Nursing, employing a Web-based platform to simulate nursing scenarios. | (B)Traditional education: regularly scheduled learning activities. |

VR, virtual reality.
